# Relationship between Obstructive Sleep Apnea and Liver Abnormalities in Older Patients: A Cross-Sectional Study

**DOI:** 10.1155/2023/9310588

**Published:** 2023-01-03

**Authors:** Yong-Xu Jin, Bi-ying Wang, Xiao-li Wang, Xing Yu, Li-da Chen, Yi-song Yang, Jie-feng Huang

**Affiliations:** ^1^Department of Respiratory and Critical Care Medicine, The First Affiliated Hospital of Fujian Medical University, Fujian Provincial Sleep-Disordered Breathing Clinic Center, Institute of Respiratory Disease, Fujian Medical University, Fuzhou 350005, China; ^2^Department of Respiratory and Critical Care Medicine, National Regional Medical Center, Binhai Campus of The First Affiliated Hospital, Fujian Medical University, Fuzhou 350212, China; ^3^Department of Pediatrics, Fujian Provincial Hospital, Gulou District, Fuzhou, Fujian 350001, China; ^4^Department of Respiratory and Critical Care Medicine, Zhangzhou Affiliated Hospital of Fujian Medical University, Xiangcheng, Zhangzhou 363000, China

## Abstract

**Background:**

Older age is a risk factor for obstructive sleep apnea (OSA), which is associated with the development of nonalcoholic fatty liver disease (NAFLD). We aimed to investigate the correlation between OSA and liver injury among older patients. *Study Design*. This is a cross-sectional study.

**Methods:**

Consecutive older (≥60 years) snoring patients were included. Subjects were divided into no OSA, mild OSA, moderate OSA, and severe OSA groups according to the apnea–hypopnea index (AHI) and were also separated into liver injury and nonliver injury groups based on liver function. Logistic regression analysis was applied to analyze the independent risk factors for liver injury.

**Results:**

We studied 227 patients (155 male, 72 female). The prevalence of liver injury exhibited an increasing trend among groups with mild-to-severe OSA. In addition, body mass index, AHI, and TG showed significant differences between the liver injury and nonliver injury groups. Logistic regression analysis revealed that AHI and TG were the major contributing factors for liver injury in older patients (adjusted odds ratio [OR] = 1.055, *p*=0.013, and OR = 1.485, *p*=0.039, respectively).

**Conclusions:**

Older patients with OSA have an increased risk of liver injury and NAFLD, and sleep apnea and high TG are important factors in contributing to the development of liver injury.

## 1. Introduction

Obstructive sleep apnea (OSA) comprises a group of chronic sleep-related breathing disorders characterized by the repeated occurrence of partial or complete upper airway obstruction during sleep. The potential consequences of OSA include cardiovascular disease [1, 2], metabolic syndrome [3, 4], neurocognitive dysfunction [5], and diminished quality of life [6]. These consequences are related to higher mortality in patients and are associated with higher healthcare costs and utilization. Compared with younger people, older people have a higher prevalence, which varied in the range of 13% to 44% [7, 8] because of the increased collapsibility of the upper airway, leading to the occurrence of obstructive apnea during sleep [9]. The prevalence of cardiovascular and cerebrovascular diseases is higher in older men with OSA, which further enhances the risk of hospitalization and health burden [10]. Therefore, it is significant to focus on older adults with OSA and its comorbidities.

Nonalcoholic fatty liver disease (NAFLD) is increasingly recognized as a common cause of chronic liver disease worldwide, representing a clinical spectrum of liver disease ranging from nonalcoholic fatty liver (NAFL) and nonalcoholic steatohepatitis (NASH) to cirrhosis and hepatocellular carcinoma [11]. A study conducted by Noureddi et al. [12] found that older NAFLD patients had a higher prevalence of NASH compared with younger patients (72% vs. 56%). Furthermore, older patients with NAFLD had higher rates of advanced fibrosis compared with younger patients with NAFLD, revealing that aging is an independent risk factor for NAFLD. It is also noteworthy that OSA may be another contributor to NAFLD development [13]. Kallwitz et al. [14] observed that the severity of OSA was related to increased alanine aminotransferase (ALT) levels and also suggested that the apnea–hypopnea index (AHI) was a major contributing factor to abnormal levels of ALT or aspartate transaminase (AST), but AHI was not an independent risk factor for NASH [15].

There has been little research concerning the correlation between OSA and liver injury as defined by hepatic enzymes in older adults. Our intent was to investigate the characteristics associated with liver injury in older patients with OSA and the influence of OSA on liver injury in these patients.

## 2. Materials and Methods

### 2.1. Study Design and Participants

Our investigation was designed as a cross-sectional study. Older subjects (≥60 years) who attended our sleep laboratory because of symptoms of sleep apnea between January 2015 and October 2019 were recruited. We excluded patients who had previously been diagnosed with OSA or used continuous positive airway pressure (CPAP). We also excluded those patients with: (1) absence of liver ultrasound examination; (2) history of secondary fatty liver disease or chronic liver disease including excess alcohol consumption (defined as >20 g/day for men and 10 g/day for women), viral hepatitis, use of liver-damaging drugs, or other causes of chronic liver disease; and (3) documented history of other diseases such as serious cardiopulmonary or renal disease or acute inflammatory disease. The study was approved by the ethics committee of the First Affiliated Hospital of Fujian Medical University (Fuzhou, China), and written informed consent was obtained from all patients.

### 2.2. Clinical and Laboratory Data

Details on clinical and biochemical measurements of all subjects were collected: history of medication (including lipid-lowering drugs, hypoglycemic drugs, and antihypertensive agents), smoking habits, and alcohol consumption. Each subject underwent measurements of weight, height, and waist circumference after the removal of shoes and a heavy coat. Body mass index (BMI) was calculated as weight in kilograms divided by the square of height in meters, and waist circumference was determined at the midpoint between the inferior costal margin and the iliac crest.

Venous blood was drawn in the morning after an overnight fast. The lipid and lipoprotein profile included total cholesterol (TC), triglycerides (TG), high-density lipoprotein-cholesterol (HDL-C), and low-density lipoprotein-cholesterol (LDL-C). The liver enzymes ALT and AST were assayed. All biochemical blood measurements were analyzed using standard laboratory procedures on the Modular P800 autoanalyzer (Roche, Tokyo, Japan).

### 2.3. Polysomnographical Evaluation

The diagnosis of OSA was established by full-night polysomnography (P-Series Sleep System, Compumedics, Victoria, Australia) including the following parameters: electrooculography, electroencephalography, electromyography, electrocardiography, snoring, oronasal airflow, thoracic and abdominal respiratory efforts, pulse oxygen saturation, and body position. The main independent variables were AHI, defined as the number of episodes of apnea and hypopnea per hour of sleep, and three different indices of nocturnal hypoxemia: the defined mean arterial oxyhemoglobin saturation during sleep as measured by blood analysis (SaO_2_), the 3% oxygen desaturation index (ODI), and the percent of sleep time with SaO_2_ < 90%. The following commonly used cut-offs for AHI were used to assess the severity of OSA: <5 events/h (no OSA), 5–15 events/h (mild OSA), >15–30 events/h (moderate OSA), and >30 events/h (severe OSA).

### 2.4. Definition of the Liver Injury and NAFLD

According to the National Health and Nutrition Examination Surveys III (NHANES III) laboratory standard [16], elevated ALT is defined as >40 U/L for males and >31 U/L for females, and elevated AST is defined as >37 U/L for males and >31 U/L for females. Liver injury is defined as elevated ALT or elevated AST. An abdominal ultrasound examination was carried out by an experienced ultrasonographer using the Technos DU8 sonography system with a 3.5 MHz transducer (Esaote SpA, Genoa, Italy). The diagnosis of NAFLD was established based on the criteria developed by the Chinese Liver Disease Association [17].

### 2.5. Statistical Analysis

All analyses were conducted using IBM SPSS Statistics for Windows, version 20.0 (IBM Corp., Armonk, NY, USA). First, we assessed whether the data were normally distributed using the Kolmogorov–Smirnov test. Continuous variables were expressed as mean ± standard deviation (SD) or median and interquartile range (IQR), depending on the distribution of data, and categorical data were presented as a number (%). Next, between-group comparisons were made for continuous data using Student's *t*-test or the Mann–Whitney U-test, and one-way analysis of variance (ANOVA) or Kruskal–Wallis H (K) for multiple-group comparison. The chi-squared test was performed for categorical variables. Last, a logistic regression model was applied to determine the dependent risk factors for NAFLD in older patients with OSA. A *p* value <0.05 was accepted as indicative of significant differences.

## 3. Results

### 3.1. Clinical Characteristics of the Study Population

Overall, 227 patients (155 male and 72 female) were enrolled in our study. The mean age was 68.0 years, and mean BMI was 26.7 kg/m^2^. Of these patients, liver injury and NAFLD were present in 18.5% and 66.1% of the entire cohort, respectively, and in 18.9% and 69.2% of the patients with OSA, respectively.

### 3.2. Comparison of the Main Clinical and Biochemical Characteristics of Patients with Respect to OSA Severity

The primary clinical and biochemical characteristics according to the severity of OSA are compared in Tables 1 and 2. Clinical characteristics including age, sex ratio, medical history, and current smoking/drinking did not differ between the four groups (no OSA, mild OSA, moderate OSA, and severe OSA). BMI, neck circumference, waist circumference, AHI, ODI, T90%, and Epworth Sleepiness Score (ESS) increased while the lowest oxygen saturation (LaSO_2_) decreased with increasing severity of OSA. The prevalence of NAFLD in the no OSA, mild OSA, moderate OSA, and severe OSA groups was 42.31%, 79.20%, 62.70%, and 68.09%, respectively, and the difference was statistically significant (*p*=0.012). Furthermore, the severe OSA group had greater prevalence of liver injury (23.4%) compared with the no OSA group (15.4%), mild OSA (10.4%), and moderate OSA group (18.6%), although this was not statistically significant.

### 3.3. Comparison of the Main Clinical and Biochemical Characteristics of Patients with Respect to Liver Injury Status

**Figure 1:**
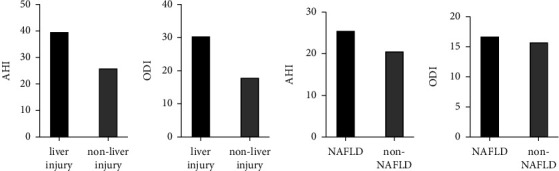
AHI and ODI alteration in NAFLD and liver injury (a), (b). The median of the apnea-hypopnea index (AHI) and oxygen desaturation index (ODI) is presented in liver injury versus non-liver injury participants. (c), (d) The median of AHI and ODI is presented in NAFLD versus non-NAFLD participants.

The primary clinical and biochemical characteristics according to the liver injury status are compared in Tables 3 and 4. Of the total 227 participants, 201 were patients with OSA (88.55%), including 38 with liver injury and 163 without liver injury. Patients with liver injury tended to be heavier and had significantly higher AHI values when compared with the nonliver injury group (BMI, 28.27 ± 4.16 kg/m^2^ vs. 26.85 ± 3.69 kg/m^2^, *p*=0.039, median of AHI 39.85 events/h vs. 26.10 events/h, *p*=0.006). They also had higher TG, gamma-glutamyl transferase, uric acid, and liver enzyme levels. The two groups did not differ significantly in terms of age, sex, neck circumference, waist circumference, ODI, T90%, LaSO_2_, and ESS. Moreover, the alterations of AHI and ODI based on the status of liver injury (median of AHI 39.85 events/h vs. 26.10 events/h, *p*=0.006, median of ODI 30.55 events/h vs. 18.05 events/h, *p*=0.060) were more obviously different than they based on the status of NAFLD group (median of AHI 25.60 events/h vs. 20.70 events/h, *p*=0.037, median of ODI 16.75 events/h vs. 15.80 events/h, *p*=0.523) (Figure 1).

### 3.4. Independent Predictors of Liver Injury and NAFLD in Patients with OSA

We performed a stepwise logistic regression analysis and found AHI (adjusted odds ratio [OR] = 1.055, *p*=0.013) and TG (OR = 1.485, *p*=0.039) were the only two variables that showed statistical significance for liver injury after adjusting for confounders including BMI, ODI, waist circumference, ESS score. In addition, BMI (OR = 1.319, *p* < 0.001), AHI (OR = 1.058, *p*=0.007), ODI (OR = 0.940, *p*=0.007) and TG (OR = 1.969, *p*=0.021) were the predictors for NAFLD.

## 4. Discussion

Our study suggests that the severity of OSA and level of TG were independently associated with liver injury in older patients with OSA. The prevalence of liver injury in the severe OSA group was higher than in the non-OSA group, and the AHI was significantly higher in the patients with liver injury. Logistic analysis showed that increased AHI was a significant risk factor for liver injury in older patients with OSA.

The prevalence of NAFLD has been increasing rapidly, especially in older individuals, which has the potential to seriously affect the health and quality of life of these patients [18]. Many studies report that OSA is an independent risk factor for NAFLD, leading to an increasing clinical burden. OSA and NAFLD may manifest with phenotypic traits in common, such as obesity and insulin resistance. A “two-hit hypothesis” suggests that insulin resistance acts as a “first hit” to result in hepatic steatosis, and oxidative injury acts as a “second hit” involved in the development of NAFLD in OSA [19]. A minority of studies [20, 21] have confirmed that biochemical evidence of liver injury (including ALT and AST) increased with the aggravation of OSA, indicating that NAFLD might happen in the setting of OSA.

In a study by Turkay et al., nearly 67% of patients referred for a clinical sleep study looking for OSA had NAFLD [22]. A cohort study investigated about 1,300 subjects with suspected OSA and found that patients with severe OSA have a 2.5-fold higher risk for liver fibrosis [23]. Our study enrolled older patients and showed the prevalence of NAFLD in OSA (69.15%) was higher than in the no-OSA group (42.31%). The incidence of liver injury (23.4%) was highest in the severe OSA group.

Many studies have shown that AHI is an independent predictor for NAFLD [24, 25]. Mesarwi et al. [25] examined 35 subjects undergoing bariatric surgery and found that the AHI was higher in those with fibrosis, and severe OSA was more prevalent among those with fibrosis. In line with previous studies, we found that AHI was the independent risk factors for NAFLD. Furthermore, our study also showed that AHI and TG were independent risk factors for liver injury in older patients with OSA. The study of Kallwitz et al. [14] enrolled 85 obese adults who underwent bariatric surgery and found OSAHS was independently correlated with the increase of ALT. Another study also supported the correlation between OSAHS and liver injury in severely obese adults who underwent weight-loss surgery. In this study, 46.5% of cases showed elevated liver enzymes, and the more severe the condition of OSAHS, the higher the probability of elevated liver enzymes. Multivariate regression showed that OSAHS and men were independently associated with elevated liver enzymes after excluding confounders [15]. However, the association between severity and liver injury was mild. The reason might involve the fact that we included elderly patients, among whom aging is more likely to be associated with liver injury. In addition, it is notable that our study did not find a link between intermittent hypoxia and liver injury, and we assume that sleep fragmentation reflected by the index of AHI inducing insulin resistance and adipose tissue inflammation might also be involved in liver injury in older patients with OSA [26, 27]. Further research is necessary to characterize the mechanisms of this relationship.

In addition, it is generally believed that obesity is a strong risk factor for the development of NAFLD. Studies investigating the prevalence of NAFLD in those patients with OSA whose mean BMI was more than 30 kg/m^2^ suggest the prevalence of OSA-complicated NAFLD was about 73%–83% [23, 28]. However, our studies did not exhibit the relationship between obesity and liver injury in older patients with OSA. Several explanations might contribute to the result. First, it has been reported that BMI does not demonstrate the same strength of association with NAFLD in older compared with middle-aged adults as the prevalence of obesity in older individuals is lower than in middle-aged adults [29]. Second, studies confirmed that aging plays an important role in inducing NAFLD by increasing lipid accumulation in the liver [30]. Further, OSA-related sleep fragmentation might also play a crucial role in NAFLD and liver injury. Taken together, the potential contribution of aging in OSA to the development of liver injury warrants further study.

Several limitations of our study have to be addressed. First, the study design itself was a cross-sectional design which only suggests a correlation but is insufficient to illustrate a cause–effect relationship. Second, the sample was relatively small because we had only recruited older subjects. Last, the diagnosis of NAFLD was based on liver ultrasonography instead of liver biopsy, which has inevitable limitations because of its low sensitivity for mild steatosis. However, ultrasonographic examination is widely used to screen for NAFLD which enables noninvasive, reliable, and accurate detection of mild-to-moderate hepatic steatosis compared to histopathologic examination. In addition, we also examined the liver function to assess liver injury.

## 5. Conclusions

In this study, we found that older patients with OSA had an increased risk of NAFLD and liver injury, and liver injury was linked with sleep apnea and high TG independently of several potential confounding factors. More attention should be given to older patients with OSA who have NAFLD and liver injuries.

## Figures and Tables

**Table 1:** Comparison of main clinical characteristics of subjects according to the severity of OSA.

	No OSA	Mild OSA	Moderate OSA	Severe OSA	*p*
Subjects (*n*)	26	48	59	94	
Age (years)	67.19 ± 6.07	69.29 ± 7.84	68.02 ± 6.48	67.59 ± 6.24	0.466
Male sex, number (%)	18 (69.2)	34 (70.8%)	37 (62.7)	66 (70.2)	0.762
HP, number (%)	14 (53.8)	36 (75.0)	42 (71.2)	68 (72.3)	0.141
DM, number (%)	4 (15.4)	16 (33.3)	20 (33.9)	25 (26.6)	0.291
Hyperlipidemia, number (%)	2 (7.7)	5 (10.4)	9 (15.3)	15 (16.0)	0.623
Antilipemic agents, number (%)	2 (7.7)	4 (8.3)	4 (6.8)	11 (11.7)	0.747
^a^ Current smoking, number (%)	5 (18.5)	11 (22.9)	12 (20.3)	17 (18.1)	0.206
Drinking	5 (19.2)	4 (8.3)	8 (13.6)	8 (8.5)	0.371
Body mass index (kg/m^2^)	23.46 ± 2.79 ^*∗*^, ^*∗∗*^, ^*∗∗∗*^	26.59 ± 3.32 ^*∗*^	26.94 ± 3.38 ^*∗∗*^	27.48 ± 4.24 ^*∗∗∗*^	<0.001
Neck circumference (cm)	36.23 ± 2.55	39.12 ± 3.78	38.30 ± 3.56	39.28 ± 3.72	0.002
Waist circumference (cm)	88.83 ± 9.21	98.56 ± 11.16	97.28 ± 10.31	98.60 ± 15.45	0.008
AHI	2.25 (1.30–3.50) ^*∗*^, ^*∗∗*^, ^*∗∗∗*^	9.70 (7.63–12.30) ^*∗*^, ^*∗*^ ^*∗*^ ^*∗*^ ^*∗*^, ^*∗*^ ^*∗*^ ^*∗*^ ^*∗*^ ^*∗*^	20.40 (17.80–25.30) ^*∗∗*^, ^*∗*^ ^*∗*^ ^*∗*^ ^*∗*^, ^*∗*^ ^*∗*^ ^*∗*^ ^*∗*^ ^*∗*^ ^*∗*^	46.34 (37.10–55.60) ^*∗∗∗*^, ^*∗*^ ^*∗*^ ^*∗*^ ^*∗*^ ^*∗*^, ^*∗*^ ^*∗*^ ^*∗*^ ^*∗*^ ^*∗*^ ^*∗*^	<0.001
ODI	2.25 (1.18–4.35) ^*∗*^, ^*∗∗*^, ^*∗∗∗*^	6.97 (4.83–9.90) ^*∗*^, ^*∗*^ ^*∗*^ ^*∗*^ ^*∗*^, ^*∗*^ ^*∗*^ ^*∗*^ ^*∗*^ ^*∗*^	14.70 (10.20–19.00) ^*∗∗*^, ^*∗*^ ^*∗*^ ^*∗*^ ^*∗*^, ^*∗*^ ^*∗*^ ^*∗*^ ^*∗*^ ^*∗*^ ^*∗*^	36.10 (27.55–47.25) ^*∗∗∗*^, ^*∗*^ ^*∗*^ ^*∗*^ ^*∗*^ ^*∗*^, ^*∗*^ ^*∗*^ ^*∗*^ ^*∗*^ ^*∗*^ ^*∗*^	<0.001
T90% (%)	0.25 (0.02–1.10) ^*∗*^, ^*∗∗*^, ^*∗∗∗*^	1.83 (0.60–8.41) ^*∗*^, ^*∗*^ ^*∗*^ ^*∗*^ ^*∗*^, ^*∗*^ ^*∗*^ ^*∗*^ ^*∗*^ ^*∗*^	4.62 (1.28–16.41) ^*∗∗*^, ^*∗*^ ^*∗*^ ^*∗*^ ^*∗*^, ^*∗*^ ^*∗*^ ^*∗*^ ^*∗*^ ^*∗*^ ^*∗*^	23.78 (8.18–45.73) ^*∗∗∗*^, ^*∗*^ ^*∗*^ ^*∗*^ ^*∗*^ ^*∗*^, ^*∗*^ ^*∗*^ ^*∗*^ ^*∗*^ ^*∗*^ ^*∗*^	<0.001
LaSO_2_ (%)	87.50 (81.00–90.00) ^*∗∗*^, ^*∗∗∗*^	84.00 (76.50–86.00) ^*∗*^ ^*∗*^ ^*∗*^ ^*∗*^ ^*∗*^	81.00 (72.00–85.00) ^*∗∗*^, ^*∗*^ ^*∗*^ ^*∗*^ ^*∗*^ ^*∗*^ ^*∗*^	75.00 (63.50–80.00) ^*∗∗∗*^, ^*∗*^ ^*∗*^ ^*∗*^ ^*∗*^ ^*∗*^, ^*∗*^ ^*∗*^ ^*∗*^ ^*∗*^ ^*∗*^ ^*∗*^	<0.001
ESS score	5.21 ± 3.44 ^*∗∗∗*^	6.78 ± 5.87	7.06 ± 4.10 ^*∗*^ ^*∗*^ ^*∗*^ ^*∗*^ ^*∗*^ ^*∗*^	9.63 ± 5.17 ^*∗∗∗*^, ^*∗*^ ^*∗*^ ^*∗*^ ^*∗*^ ^*∗*^ ^*∗*^	<0.001

^a^ Current smoker was the one who had a smoking history and had not quit smoking. OSA, obstructive sleep apnea; HP, hypertension; DM, diabetes mellitus; BMI, body mass index; AHI, apnea-hypopnea index; ODI, oxygen desaturation index; T90%, the percentage of total sleep time spent with SpO_2_ < 90%, LaSO_2_ lowest O_2_ saturation; ESS score, Epworth sleepiness scale score.  ^*∗*^: if *p*  <  0.05 between no OSA group and mild OSA group;  ^*∗∗*^: if *p*  <  0.05 between no OSA group and moderate OSA;  ^*∗∗∗*^: if *p*  <  0.05 between no OSA group and severe OSA group;  ^*∗*^ ^*∗*^ ^*∗*^ ^*∗*^: if *p*  <  0.05 between mild OSA group and moderate OSA group;  ^*∗*^ ^*∗*^ ^*∗*^ ^*∗*^ ^*∗*^: if *p*  <  0.05 between mild OSA group and severe OSA group;  ^*∗*^ ^*∗*^ ^*∗*^ ^*∗*^ ^*∗*^ ^*∗*^: if *p*  <  0.05 between moderate OSA group and severe OSA group.

**Table 2:** Comparison of biochemical characteristics of subjects according to the severity of OSA.

	No OSA	Mild OSA	Moderate OSA	Severe OSA	*p*
TC (mmol/L)	4.48 ± 0.99	4.22 ± 1.17	4.64 ± 1.06	4.42 ± 1.07	0.258
TG (mmol/L)	1.14 (0.94–1.66)	1.38 (0.98–1.85)	1.33 (1.08–1.98)	1.36 (0.97–2.01)	0.717
HDL-C (mmol/L)	1.15 ± 0.31	1.09 ± 0.32	1.09 ± 0.29	1.08 ± 0.25	0.773
LDL-C (mmol/L)	2.91 ± 0.78	2.55 ± 1.02	2.96 ± 0.0.77	2.85 ± 0.88	0.104
Fasting glucose (mmol/L)	5.02 (4.59–5.58)	5.23 (4.67–6.44)	5.25 (4.63–6.07)	5.26 (4.58–6.01)	0.558
Creatinine (*μ*mol/L)	71.03 ± 19.88	72.13 ± 20.38	68.50 ± 18.09	75.90 ± 40.66	0.515
BUN (mmol/L)	4.99 ± 1.48	5.67 ± 1.77	5.37 ± 1.31	5.72 ± 2.64	0.389
ALP (U/L)	70.16 ± 13.33	81.23 ± 50.27	70.93 ± 29.18	68.03 ± 19.13	0.107
GGT (mmol/L)	23.50 (15.75–37.10)	33.00 (23.25–54.75)	28.00 (19.00–45.00)	27.00 (20.75–44.00)	0.127
T. bilirubin (umol/L)	10.00 (8.03–14.85)	10.55 (7.88–14.25)	11.40 (9.20–14.00)	10.25 (7.65–13.90)	0.758
ALT (U/L)	17.50 (12.78–28.50)	21.50 (17.00–26.00)	20.00 (15.00–30.00)	21.00 (17.00–32.00)	0.213
AST (U/L)	19.80 (15.98–25.25)	20.00 (16.00–23.75)	29.00 (16.00–26.00)	21.75 (17.00–27.00)	0.483
Uric acid (*μ*mol/L)	352.65 (275.55–457.50)	348.00 (283.75–413.78)	359.80 (273.30–420.00)	360.00 (307.35–434.50)	0.383
NAFLD, number (%)	11 (42.31) ^*∗*^	38 (79.20) ^*∗*^	37 (62.70)	64 (68.09)	0.012
Elevated ALT^a^, number (%)	4 (15.4)	4 (8.3)	9 (15.3)	20 (21.3)	0.261
Elevated AST^a^, number (%)	1 (3.8)	3 (6.3)	9 (15.3)	11 (11.7)	0.297
Liver injury, number (%)	4 (15.4)	5 (10.4)	11 (18.6)	22 (23.4)	0.290

OSA, obstructive sleep apnea; TC, total cholesterol; TG, triglycerides; HDL-C, high-density lipoprotein-cholesterol; LDL-C, low-density lipoprotein-cholesterol, BUN, blood urea nitrogen; ALP, alkaline phosphatase; GGT, gamma-glutamyltransferase; ALT, alanine aminotransferase; AST, aspartate aminotransferase; T. bilirubin total bilirubin; NAFLD, nonalcoholic fatty liver disease.  ^*∗*^: if *p*  <  0.05 between no OSA group and mild OSA group;  ^*∗∗*^: if *p*  <  0.05 between no OSA group and moderate OSA;  ^*∗∗∗*^: if *p*  <  0.05 between no OSA group and severe OSA group;  ^*∗*^ ^*∗*^ ^*∗*^ ^*∗*^: if *p*  <  0.05 between mild OSA group and moderate OSA group;  ^*∗*^ ^*∗*^ ^*∗*^ ^*∗*^ ^*∗*^: if *p*  <  0.05 between mild OSA group and severe OSA group;  ^*∗*^ ^*∗*^ ^*∗*^ ^*∗*^ ^*∗*^ ^*∗*^: if *p*  <  0.05 between moderate OSA group and severe OSA group.

**Table 3:** Comparison of the main clinical of OSA patients according to liver injury status.

	Nonliver injury	Liver injury	*p*
Subjects (*n*)	163	38	
Age (years)	68.41 ± 7.01	66.90 ± 5.24	0.213
Male sex, number (%)	114 (69.9)	23 (60.5)	0.262
HP, number (%)	117 (71.8)	29 (76.3)	0.572
DM, number (%)	52 (31.9)	9 (23.7)	0.321
Hyperlipidemia, number (%)	20 (12.3)	9 (23.7)	0.071
Antilipemic agents, number (%)	17 (10.4)	2 (5.3)	0.538
Current smoking^a^, number (%)	31 (19.0)	9 (23.7)	0.517
Drinking	16 (9.8)	4 (15.0)	0895
Body mass index (kg/m^2^)	26.85 ± 3.69	28.27 ± 4.16	0.039
Neck circumference (cm)	38.95 ± 3.82	38.92 ± 3.16	0954
Waist circumference (cm)	97.66 ± 13.59	100.55 ± 10.21	0.225
AHI	26.10 (14.30–39.90)	39.85 (19.83–54.05)	0.006
ODI	18.05 (9.48–34.05)	30.55 (11.18–43.04)	0.060
T90% (%)	7.75 (1.68–28.03)	17.50 (2.35–51.53)	0.076
LaSO_2_ (%)	79.00 (70.00–84.00)	77.00 (68.25–84.00)	0.322
ESS score	7.90 ± 5.13	9.33 ± 5.38	0.140

^a^ Current smoker was the one who had a smoking history and had not quit smoking. OSA, obstructive sleep apnea; HP, hypertension DM diabetes mellitus; BMI, body mass index; AHI, apnea-hypopnea index; ODI, oxygen desaturation index; T90%, the percentage of total sleep time spent with SpO_2_ < 90%; LaSO_2_, lowest O_2_ saturation; ESS score, Epworth sleepiness scale score.

**Table 4:** Comparison of biochemical characteristics of OSA patients according to liver injury status.

	Nonliver injury	Liver injury	*p*
TC (mmol/L)	4.42 ± 1.11	4.47 ± 1.05	0.822
TG (mmol/L)	1.28 (0.97–1.82)	1.75 (1.33–2.24)	0.003
HDL-C (mmol/L)	1.10 ± 0.29	1.04 ± 0.25	0228
LDL-C (mmol/L)	2.80 ± 0.90	2.88 ± 0.84	0.597
Fasting glucose (mmol/L)	5.23 (4.62–6.08)	5.30 (4.59–6.41)	0.734
Creatinine (*μ*mol/L)	71.56 ± 31.14	77.43 ± 24.59	0.572
BUN (mmol/l)	5.63 ± 2.29	5.50 ± 1.14	0.745
ALP (U/L)	72.31 ± 31.43	70.85 ± 35.91	0.608
GGT (mmol/L)	25.00 (19.00–22.00)	45.50 (38.00–70.25)	<0.001
T. bilirubin (umol/L)	10.60 (8.10–14.20)	11.65 (8.60–13.00)	0.687
ALT (U/L)	19.00 (16.00–24.00)	54.00 (36.75–62.25)	<0.001
AST (U/L)	19.00 (16.00–22.00)	37.00 (29.75–42.75)	<0.001
Uric acid (*μ*mol/L)	351.40 (282.13–413.75)	416.30 (346.33–462.50)	0.001

OSA, obstructive sleep apnea; TC, total cholesterol; TG, triglycerides; HDL-C, high-density lipoprotein-cholesterol; LDL-C, low-density lipoprotein-cholesterol; BUN, blood urea nitrogen; ALP, alkaline phosphatase; GGT, gamma-glutamyltransferase; ALT, alanine aminotransferase; AST, aspartate aminotransferase; T. bilirubin total bilirubin.

## Data Availability

The datasets used and analyzed during the current study are available from the corresponding author on reasonable request.
